# Interspinous Process Decompression: Expanding Treatment Options for Lumbar Spinal Stenosis

**DOI:** 10.1155/2016/3267307

**Published:** 2016-10-13

**Authors:** Pierce D. Nunley, A. Nick Shamie, Scott L. Blumenthal, Douglas Orndorff, Jon E. Block, Fred H. Geisler

**Affiliations:** ^1^Spine Institute of Louisiana, Shreveport, LA 71101, USA; ^2^Ronald Reagan UCLA Medical Center, Westwood, CA 90095, USA; ^3^Texas Back Institute, Plano, TX 75093, USA; ^4^Spine Colorado, Durango, CO 81301, USA; ^5^2210 Jackson Street, Suite 401, San Francisco, CA 94115, USA; ^6^401 N. Wabash Avenue, Suite 62F, Chicago, IL 60611, USA

## Abstract

Interspinous process decompression is a minimally invasive implantation procedure employing a stand-alone interspinous spacer that functions as an extension blocker to prevent compression of neural elements without direct surgical removal of tissue adjacent to the nerves. The Superion® spacer is the only FDA approved stand-alone device available in the US. It is also the only spacer approved by the CMS to be implanted in an ambulatory surgery center. We computed the within-group effect sizes from the Superion IDE trial and compared them to results extrapolated from two randomized trials of decompressive laminectomy. For the ODI, effect sizes were all* very large* (>1.0) for Superion and laminectomy at 2, 3, and 4 years. For ZCQ, the 2-year Superion symptom severity (1.26) and physical function (1.29) domains were* very large*; laminectomy effect sizes were* very large* (1.07) for symptom severity and* large* for physical function (0.80). Current projections indicate a marked increase in the number of patients with spinal stenosis. Consequently, there remains a keen interest in minimally invasive treatment options that delay or obviate the need for invasive surgical procedures, such as decompressive laminectomy or fusion. Stand-alone interspinous spacers may fill a currently unmet treatment gap in the continuum of care and help to reduce the burden of this chronic degenerative condition on the health care system.

## 1. Introduction

Lumbar spinal stenosis is a classic neural compression syndrome where spine extension causes constriction of the nerve roots exiting the spinal column. Stenotic arthritic encroachment reduces the foraminal aperture resulting in the primary patient complaint of intermittent neurogenic claudication [1]. A simple postural solution to resolve these symptoms is to move the spine into flexion thereby decompressing the nerve roots. The “gold standard” surgical option, laminectomy, decompresses the neural structures by directly removing impinging ligament and bone [2]. Over 175,000 surgeries are performed to treat spinal stenosis annually in the US, making it the number one reason for spine surgery in the elderly population [3].

As an alternative, interspinous process decompression is a minimally invasive procedure that builds on the concept that back extension is a seminal factor in the causative chain that instigates neurogenic claudication. This procedure involves the implantation of a stand-alone interspinous spacer that functions by serving as a lumbar vertebral joint extension blocker to prevent compression of neural elements in extension. The spacer blocks the extension motion without exposure or removal of tissue adjacent to the dura or exiting nerves [4].

**Figure 1:**
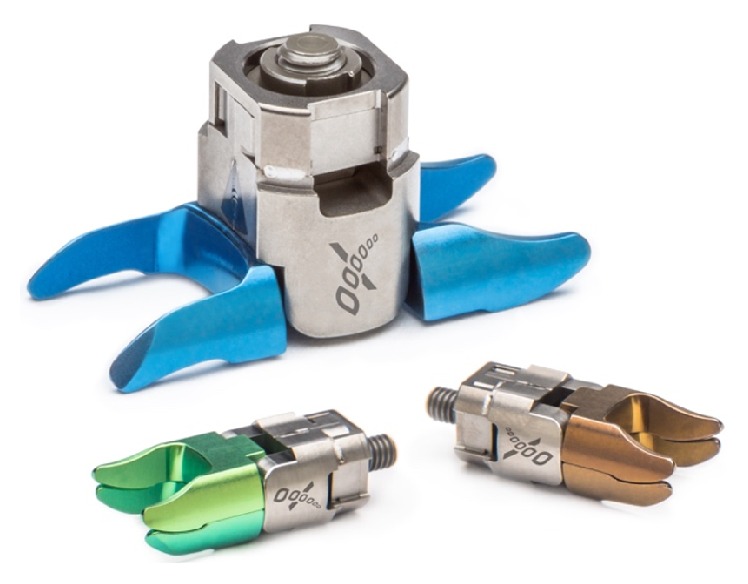
The Superion interspinous spacer.

On 20 May 2015, the US Food and Drug Administration (FDA) approved the Superion Interspinous Decompression System (VertiFlex, San Clemente, CA, USA) for commercial distribution. Not requiring concomitant surgical decompression, this second generation stand-alone interspinous device is the only spacer commercially available to physicians in the US (Figure 1). Following US regulatory approval, the Superion achieved a number of consequential and significant regulatory and clinical milestones. First, the AMA CPT® Editorial Panel approved the addition of Category I CPT codes to describe one- and two-level insertion of stand-alone interspinous spacers at their October 2015 meeting. Effective January 1, 2017, the new Category I codes will replace the existing Category III CPT codes that applied to first generation spacers. Second, the Centers for Medicare and Medicaid Services (CMS) added the insertion of interspinous spacers to their list of approved surgical procedures in Ambulatory Surgery Centers, effective January 1, 2016. Additionally, several peer reviewed publications of the Superion Investigational Device Exemption (IDE) trial have documented the large, statistically significant improvements achieved in condition-specific, pain, and functional clinical outcomes following device implantation at 6 months, 2 years, and 3 years [7–10]. Accomplishing this series of milestones substantiates the graduation of the Superion device from a concept with potential to an acceptable and practical clinical modality for the treatment of intermittent symptoms of neurogenic claudication secondary to moderate spinal stenosis.

## 2. Materials and Methods

**Figure 2:**
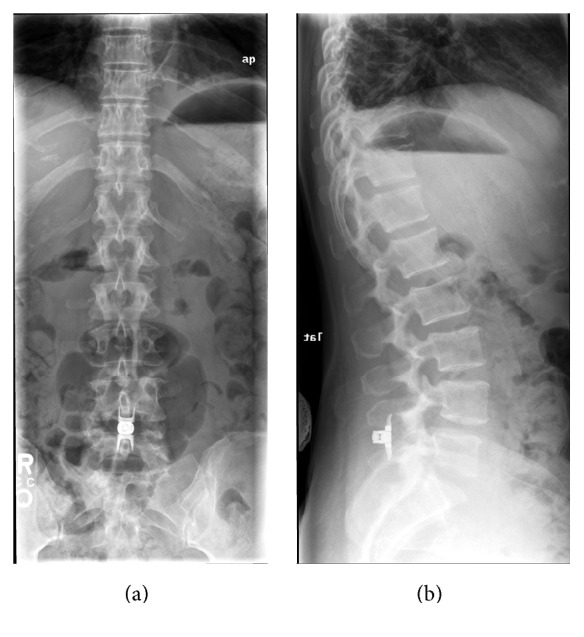
Anteroposterior (a) and lateral (b) plain radiographic images showing proper anatomical positioning of the Superion spacer* in situ*.

The Superion is indicated to treat skeletally mature patients suffering from pain, numbness, and/or cramping in the legs (intermittent neurogenic claudication) secondary to a diagnosis of moderate degenerative lumbar spinal stenosis, with or without Grade 1 spondylolisthesis, confirmed by X-ray, MRI, and/or CT evidence of thickened ligamentum flavum, narrowed lateral recess, and/or central canal or foraminal narrowing. The Superion is indicated for those patients with impaired physical function who experience relief in flexion from symptoms of leg/buttock/groin pain, numbness, and/or cramping, with or without back pain, and who have undergone at least 6 months of nonoperative treatment. The Superion may be implanted at one or two adjacent lumbar levels in patients in whom treatment is indicated at no more than two levels, from L1 to L5 (Figure 2).

For this intended use, moderate degenerative lumbar spinal stenosis is defined as follows:

- 25% to 50% reduction in the central canal and/or nerve root canal (subarticular, neuroforaminal) compared to the adjacent levels on radiographic studies, with radiographic confirmation of any one of the following:
  - Evidence of thecal sac and/or* cauda equina* compression
  - Evidence of nerve root impingement (displacement or compression) by either osseous or nonosseous elements
  - Evidence of hypertrophic facets with canal encroachment
- Also associated with the following clinical signs:
  - Present with moderately impaired physical function (PF) defined as a score of ≥2.0 of the Zurich Claudication Questionnaire (ZCQ)
  - Ability to sit for 50 minutes without pain and to walk 50 feet or more.

To gauge the practical clinical significance of the published Superion IDE findings, we computed the within-group (i.e., Superion arm only) effect size at each annual postoperative interval compared to baseline for each clinical outcome separately through 4 years using Cohen's formula and thresholds [11, 12]. The effect size is computed as the standardized difference between two means or, simply put, the mean score (preop) – mean score (follow-up)/standard deviation of the change. Effect sizes are typically reported in the range from 0.0 (no effect) to >1.0 (very large effects) with the following thresholds: 0.2 (small effect), 0.5 (medium effect), 0.8 (large effect), and >1.0 (very large effect). The effect size calculation provides some normalization for baseline and distribution imbalances.

We identified two published laminectomy studies that included at least one of the same outcomes as the Superion IDE trial and included sufficient data to compute corresponding effect sizes. First, the Superion IDE results were compared with published findings for decompressive laminectomy by extrapolating within-group 4-year effect sizes for the Oswestry Disability Index (ODI) from the published report of the NIH-sponsored SPORT trial, the largest study of surgical and nonsurgical management of lumbar stenosis [5, 13]. Similarly, we estimated the 2-year effect size for Zurich Claudication Questionnaire (ZCQ) symptom severity and physical function domains for decompressive laminectomy based on the published report of Strömqvist et al. [6].

## 3. Results

**Figure 3:**
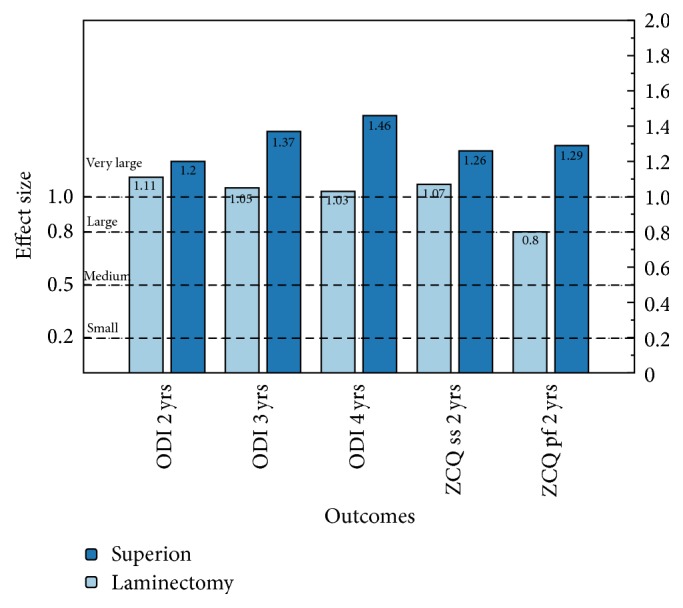
Within-group effect sizes calculated from the Superion IDE trial in contrast with comparable effect size extrapolated from the SPORT trial [5] for ODI outcomes at 2, 3, and 4 years of follow-up and from Strömqvist et al. [6] for ZCQ symptom severity (ss) and physical function (pf) at 2 years of follow-up.

The comparative within-group effect sizes for Superion and laminectomy treatments are provided in Figure 3. For back function, the ODI results consistently showed* very large* effect size estimates for both treatments at all follow-up intervals. Superion ODI effect sizes were particularly robust. For condition-specific impairment, the 2-year ZCQ results were similarly robust for Superion treatment with* very large* effect sizes for both the symptom severity and physical function domains. In contrast, while laminectomy resulted in a* very large* effect size for symptom severity, the physical function domain result just met the threshold for a* large* effect size. Overall, the effect sizes for Superion were uniformly higher than those reported for the “gold standard” treatment and did not exhibit worsening with time.

## 4. Discussion

**Figure 4:**
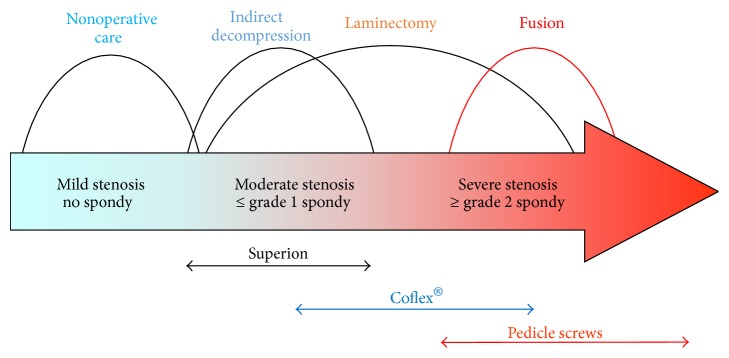
The continuum of care of treatment for lumbar spinal stenosis. Superion represents the “first line” option for minimally invasive surgical treatment.

The Superion is the second “stand-alone” interspinous spacer approved by the FDA and the only one currently available on the US market. Importantly, the implantation procedure does not cause substantial alterations or disruptions to the spinal anatomy adjacent to neural structures. Specifically, the epidural space is not surgically exposed during spacer insertion, whereas laminectomy decompression directly opens the epidural space. The surgical exposure of the epidural space is known to routinely produce epidural scar, adhesions, and tethering around the dural sac and exiting nerve roots, which can cause symptomatic problems [14, 15]. Additionally, if subsequent surgical procedures are necessary to address progressive degenerative changes and/or reemergence of symptoms, the avoidance of the epidural space in the Superion placement reduces the complexity of future surgical options compared to starting with a laminectomy procedure. Also, if device removal is required, the implant can be explanted via the same minimally invasive access as the original implantation procedure. This suggests that interspinous spacers may be considered a reasonable “first line” option in the continuum of care for the treatment of moderate lumbar spinal stenosis (Figure 4).

The minimization of iatrogenic insult associated with implantation of interspinous spacers significantly reduces the risk of operative adverse events. In a recent review of spinal devices in the Medicare population, higher perioperative complication rates were found in decompression surgeries compared to interspinous spacers [16]. Because of the minimally invasive nature of the surgery, implantation of spacers can be accomplished under local anesthesia in an ambulatory surgical setting or with conscious sedation.

Lauryssen et al. [17] documented consistently similar clinical improvements in back and leg pain, back function, and condition-specific impairment for both the Superion and decompressive laminectomy at 2 years postoperatively. Herein, we found robust effect sizes (>1.0) at each postoperative follow-up interval for ODI and ZCQ with Superion treatment, uniformly higher than comparable effect sizes with laminectomy, and the magnitude of the effect size was durable through 4 years of follow-up. While estimates of the practical clinical significance of the Superion compare favorably with published results for laminectomy, enthusiasm should be tempered by the known limitations of using historical controls. Nevertheless, these results are encouraging and support the use of the Superion as an effective treatment option for lumbar spinal stenosis.

## 5. Conclusions

Interspinous spacers fill a distinct treatment gap in the continuum of care for patients with moderate degenerative lumbar spinal stenosis. These patients have exhausted conservative care but may be inappropriate candidates for or unwilling to undergo surgical decompressive laminectomy. Because spacers are implanted in a minimally invasive fashion without anatomical disruption, they can be easily removed and converted to laminectomy if symptoms reemerge. This study corroborates previous reports that found similar clinical benefit provided by both spacers and laminectomy, providing the patient with a minimally invasive surgical option without compromising the extent or time duration of the symptom relief.
